# Giant Tubular Adenoma of the Breast in an Adolescent: Diagnostic Challenges and Literature Review

**DOI:** 10.30699/ijp.2025.2056026.3428

**Published:** 2025-07-01

**Authors:** Monica Mishra, Seetu Palo

**Affiliations:** *Department of Pathology and Laboratory Medicine, Administrative Block, All India Institute of Medical Sciences, Bibinagar, Telangana, India*

**Dear Editor,**

Tubular adenomas are rare, comprising 0.13% to 1.7% of all benign breast neoplasms (1). While the tumor size varies between 1 cm and 7.5 cm, it seldom surpasses 5 cm, with only a very few reports of ‘giant tubular adenoma’ are on the record. We present a case of giant tubular adenoma in a young female along with a brief discussion on differential diagnoses and diagnostic difficulties in differentiating them from other fibroepithelial and benign proliferative breast lesions. This case represents the third-largest giant tubular adenoma documented in an adolescent till date, with a size of 12×10×8 cm and adds to the growing but still limited body of literature in this regard. Additionally, this report consolidates previously scattered data by including a comparative summary of clinicopathological attributes of all published giant tubular adenoma cases [Table 1] (2-10).

**Fig. 1:**
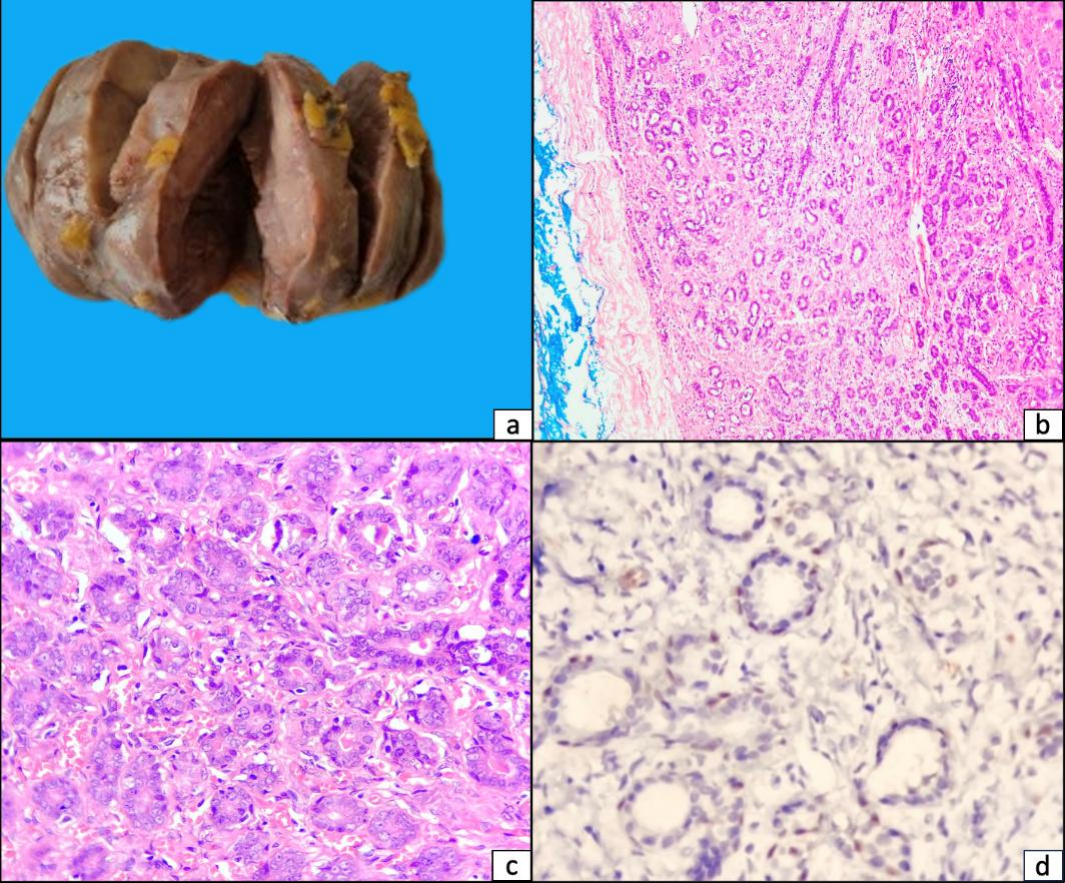
(Original): (a) Excised tan, homogenously solid mass; (b) Microphotograph showing capsulated tumor comprising of closely arranged tubules on a fibrous stroma [H&E stain, 40×]; (c) Closely packed small, uniform tubules with scant intervening stroma [H&E stain, 400×]; (d) p63 immunostaining highlighting the preserved outer myoepithelial cell layer in the tubules [IHC, 400×]

A 17-year-old female presented with gradually enlarging, non-tender lump in the right breast for over two years. Upon examination, a mobile, firm lump measuring 12×10 cm was noted. There was no axillary lymphadenopathy. Ultrasound revealed a well-defined, solid, hypoechoic lesion with no vascularity (BIRADS-III, likely fibroadenoma). Fine needle aspiration cytology was also suggestive of fibroadenoma. Lumpectomy was performed. Grossly, the mass was homogenously solid, measuring 12×10x8 cm and weighing 400 grams. Microscopy revealed tubular adenoma with closely arranged uniform glandular structures (with preserved outer myoepithelial cell layer) in scant fibrous stroma, without atypia or malignancy [Figure 1]. Post-operative period was uneventful.

**Fig. 2:**
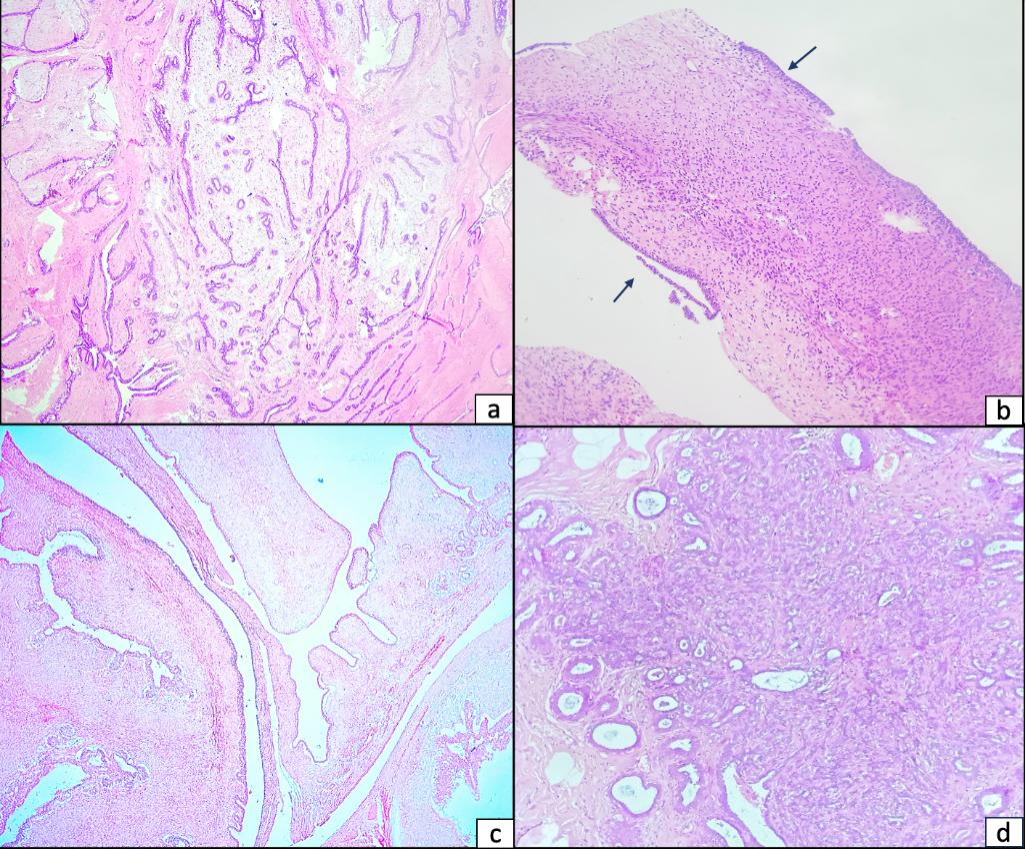
(Original): (a) Fibroadenoma displaying benign ductal structures embedded within a fibromyxoid stroma, displaying a predominantly pericanalicular growth pattern [H&E stain, 40×]; (b) Trucut biopsy of benign phyllodes showing cellular stroma with scant epithelial component (arrow) [H&E stain, 40×]; (c) Excision biopsy of benign phyllodes exhibiting broad, frond-like epithelial-lined stromal projections with cleft-like spaces [H&E stain, 40×]; (d) Sclerosing adenosis demonstrating crowded, distorted glandular elements within a sclerotic stroma [H&E stain, 100×]

Giant tubular adenomas of the breast present a diagnostic dilemma primarily due to their clinical and radiological resemblance to other breast lesions, particularly fibroadenomas and phyllodes tumors. Tubular adenomas appear similar to fibroadenomas on mammography and ultrasound, that is, as solid, well-delineated, hypoechoic masses (1,11). In some instances, they can mimic malignant masses with microcalcifications, posing diagnostic dilemma (12). In such cases, a core biopsy under image guidance is helpful in pre-operative diagnosis. Microscopically, while fibroadenomas often contain a mix of epithelial and stromal components [Figure 2a], tubular adenomas are composed of closely packed, uniform tubules with minimal stroma. Fine needle aspiration cytology cannot reliably distinguish tubular adenoma from fibroadenoma due to overlapping cytomorphological features as typified by our case as well. Sengupta et al analyzed 33 cases of tubular adenoma of the breast, wherein cytology confirmed the diagnosis in only 2 cases, while radiological evaluation failed to correctly identify any cases (12). Phyllodes tumors, especially benign or borderline forms, may mimic tubular adenomas both clinically and radiologically, as seen in the provisional diagnosis of the current case. Unlike tubular adenomas, phyllodes tumors exhibit predominance of stromal component with increased stromal cellularity [Figure 2b,c]. Clinically, phyllodes tumors carry a risk of recurrence and potential for malignant transformation, necessitating their distinction from tubular adenomas, which are benign at large.

While other benign lesions—such as lactating adenoma, ductal adenoma, nipple adenoma, microglandular adenosis, tubular adenosis and sclerosing adenosis —may enter the differential diagnosis, these are less frequently confused with giant tubular adenomas and were not prominent considerations in this case (2,11). Lactating adenomas, which share similarities with tubular adenomas under different physiological conditions, can be distinguished by the presence of prominent cytoplasmic vacuoles in the tubule-lining epithelium and abundant luminal secretions indicative of lactational changes. Microglandular adenosis is characterized by the proliferation of small, round tubules lined by a single layer of epithelial cells. This distinguishes it from tubular adenoma, as immunohistochemistry confirms the absence of myoepithelial cells in microglandular adenosis. Sclerosing adenosis can resemble tubular adenoma in morphology but typically features a more abundant and sclerosed stromal component, glandular distortion and absence of capsule [Figure 2d]. 

The only malignant lesion that closely resembles tubular adenoma is tubular carcinoma, which may appear deceptively benign histologically. Tubular carcinoma is characterized by stellate infiltrative growth pattern and absence of outer myoepithelial cell layer in the tubules (13). In diagnostically challenging cases, immunohistochemical staining for myoepithelial markers such as p63 and calponin can aid in distinguishing tubular adenoma from tubular carcinoma.

The impact of giant tubular adenomas on patient outcomes is generally positive, given the benign nature of the condition, but requires careful surgical planning to ensure satisfactory cosmetic results owing to large size. Although malignant transformation of tubular adenoma has been documented in four reported cases, the risk of carcinoma remains low (14). 

To conclude, giant tubular adenoma of the breast represents an extremely rare clinical entity, posing diagnostic challenges. Given its clinical and radiological resemblance to other fibroepithelial lesions—such as fibroadenoma and phyllodes tumor—it should be included in the differential diagnosis of large, well-circumscribed, palpable breast masses in young females. Imaging modalities such as ultrasound and fine needle aspiration cytology often lack sufficient specificity to reliably differentiate these entities due to overlapping features. Hence, core needle biopsy is preferred for establishing a definitive preoperative diagnosis for guiding appropriate surgical intervention, achieving favourable cosmetic outcomes, and avoiding unnecessary overtreatment. This case highlights the importance of histopathological confirmation for large breast masses to prevent misdiagnosis. Greater clinical awareness of this entity can aid in timely diagnosis and tailored management.

**Table 1:** Clinicopathological attributes of published cases of giant tubular adenoma of breast (3-10)

Author and year of publication	Age	Symptoms at presentation	Features at USG	Pre-operative tissue diagnosis	Size (in cm)	Follow-up	Any other remarks, if any
Dusunceli et al, 2012	43 y	Diffuse enlargement of right breast	Lump occupying the whole breast with low resistant arterial vasculature- BIRADS 5	No evidence of malignancy on core biopsy	14x13	**-**	Tubular adenoma can mimic as malignant lesions upon imaging
Haung et al, 2015	26 y	Rapid enlargement of lump in left accessory breast	Homogenously solid	-	15x15x12	**-**	Pregnancy associated
Kalipatnapu et al, 2015	23 y	Gradually increasing painless lump in left breast	-	-	9x8.5x5	-	Co-existing fibroadenoma in the adjacent parenchyma
Miller et al, 2018	29 y	Non tender right breast lump	Well-circumscribed hypoechoic mass with internal vascularity	Tubular adenoma on core biopsy	10x9.5x4	No complaints at one year post surgery	**-**
Mazingi et al, 2019	31 y	Right-sided, slow growing breast mass for 10 years, associated with surface ulceration	-	Tubular adenoma on core biopsy	18 ×15×10	No postoperative complications	Maffucci’s syndrome
Chehab et al, 2022	21 y	Lump in right breast for one year	Hypoechoic mass with microcalcification-BIRADS 4b	-	10x7x2.5	No complaints at 1.5 year post surgery	Microcalcification can be associated with tubular adenoma
Qasmi et al, 2022	18 y	Painless lump in left breast	well-circumscribed, solid, hypoechoic lesion with posterior acoustic enhancement	Fibro-epitheliallesion on core biopsy	10×10×5	No postoperative complications	-
Chang et al, 2024	14 y	Gradually progressive lump in right breast for one year	Well defined hypoechoic mass with thin internal septations	Fibroadenoma on FNAC	12.5x11.5x7	No complaints at one year post surgery	-
Present Case	17 y	Non-tender lump in the right breast for over two years	well-defined, solid, hypoechoic lesion- BIRADS 3	Fibroadenoma on FNAC	12x10x8	No complaints at one year post surgery	-
